# A Rapidly Progressive Case of Statin-induced Necrotizing Autoimmune Myopathy

**DOI:** 10.7759/cureus.7021

**Published:** 2020-02-17

**Authors:** Sundus Ahmed, Violeta Capric, Muhammad Khan, Prabash Koneru

**Affiliations:** 1 Internal Medicine, Kings County Hospital Center, Brooklyn, USA; 2 Internal Medicine, State University of New York Downstate Medical Center, Brooklyn, USA

**Keywords:** statin, myopathy, statin-induced necrotizing autoimmune myopathy, anti-hmgcr, proximal muscle weakness, rheumatology

## Abstract

Statins are one of the most commonly used medications to lower cholesterol and have been known to cause various side effects including myalgias, myopathies, and rhabdomyolysis. Statin-induced necrotizing autoimmune myopathy (SINAM), a subtype of inflammatory myopathy, is an exceedingly rare but severe side effect of statin use that manifests as progressive muscle weakness. We describe a rapidly progressive case of SINAM in a 66-year-old Haitian female who developed debilitating symptoms after one month of statin use. Despite aggressive treatment with steroids and immunosuppressants, she failed to regain muscle strength and functional status, and remains on therapy. Given the widespread use of statins, it is important for clinicians to be aware of this condition and its presenting symptoms in order to initiate prompt treatment.

## Introduction

Statin-induced necrotizing autoimmune myopathy (SINAM) is a rare type of inflammatory myopathy characterized by widespread muscle necrosis and antibodies against 3-hydroxy-3-methylglutaryl-coenzyme A reductase (HMGCR), a key enzyme in cholesterol synthesis that is also the site of statin inhibition. It typically presents with proximal muscle weakness and an elevated creatine kinase (CK), findings that are also common in other autoimmune myopathies. Diagnosis thus requires a high index of suspicion and is especially likely when discontinuation of the statin leads to no improvement in symptoms. We present a case of SINAM in a patient who developed debilitating symptoms one month after initiating atorvastatin therapy.

## Case presentation

A 66-year-old Haitian female with a history of diabetes mellitus and sickle cell trait presented with one week of proximal muscle weakness and myalgias (most prominent in the upper arms and legs) after having started atorvastatin 40 mg one month prior (indicated for hyperlipidemia in the setting of diabetes). Physical exam showed muscle strength of 5/5 in all extremities and normal gait; however, pain was reproducible on palpation of the proximal muscles in both the upper and lower extremities. No obvious joint swelling or tenderness was found, and vitals were within normal limits. Laboratory tests were significant for an elevated CK of 16,252 U/L (N: 25-170 U/L), aspartate aminotransferase (AST) of 551 (N: 10-40 U/L), alanine aminotransferase (ALT) of 602 (N: 10-45 U/L), and 2+ hemoglobin (HgB) without red blood cells (RBCs) on urinalysis (i.e. myoglobinuria). The patient was suspected to have rhabdomyolysis, and the treatment was initiated with aggressive fluid hydration and discontinuation of the statin. She was discharged three days later upon downtrending of her CK.

The patient presented again two weeks later with rapidly progressing proximal muscle weakness. She endorsed difficulty standing from a seated position, ambulating, and holding her head up without support, and a new-onset dysphagia to both solids and liquids. Physical exam was significant for diminished muscle strength of 3/5 in the proximal muscles and 4/5 in the distal muscles bilaterally in both the upper and lower extremities, and gait was noted to be wide based and cautious. Laboratory tests showed an increasingly elevated CK of 18,310 U/L (N: 25-170 U/L), an AST of 709 (N: 10-40 U/L), an ALT of 897 (N: 10-45 U/L), an elevated C-reactive protein of 56 mg/L (N: <3 mg/L), and an elevated troponin of 0.733 ng/mL (N: <0.4 ng/mL, without electrocardiographic changes or chest pain). Urinalysis was significant for 3+ protein and Hgb without RBCs. Thyroid function tests were within normal limits, and HIV testing was negative. The patient was admitted for an inability to ambulate and myositis of unknown etiology, and aggressive fluid hydration was provided. Additional laboratory studies were performed to rule out autoimmune myositis, and the patient underwent a bedside ultrasound which showed diffuse edema in the rectus femoris muscle. Muscle biopsy of this site showed widespread myofiber necrosis, and an electromyography was significant for irritative myopathy. Laboratory studies showed negative antinuclear antibody (ANA) and anti-Jo-1 antibodies and strongly positive anti-HMGCR antibodies at >200 (N: 0-19), after which a diagnosis of SINAM was made.

Treatment was initiated with methylprednisolone 1 g/day (tapered to oral prednisone 40 mg/day over one week), which caused a rapid decline in the patient’s CK but no improvement in muscle strength. Barium esophagram and videofluoroscopy were performed due to worsening symptoms and showed that the patient had severe esophageal dysmotility, at which point dietary modifications were provided. Due to a lack of improvement, the patient then received a five-day course of intravenous immunoglobulin (IVIG). Muscle strength and functional status continued to decline, requiring nasogastric tube for feeds. The patient was transferred to the intensive care unit for close monitoring and impending respiratory failure. Due to poor response to therapy, the patient underwent five days of plasma exchange, rituximab infusion, and azathioprine 50 mg/day. Despite her CK decreasing to 415 U/L, no improvement was seen in muscle strength. The patient was discharged on azathioprine 150 mg/day, tacrolimus 4 mg/day, and prednisone 5 mg/day after a total seven-week hospital stay.

## Discussion

Since their introduction in 1982, statins (3-hydroxy-3-methylglutaryl-coenzyme A reductase inhibitors) have become one of the most commonly prescribed medications worldwide [1]. Deemed the only lipid-lowering agents causing a significant decrease in mortality from cardiovascular disease by the American College of Cardiology/American Heart Association Blooddown Cholesterol Treatment Task Force, statins are currently recommended for an estimated 56 million U.S. adults (almost 50% of the total population) [2,3].

Although generally well tolerated, statins have been associated with a number of skeletal muscle-related side effects including self-limiting myalgias, symptomatic myopathy, and rhabdomyolysis [4]. Interestingly, Caribbean and Black Africans were found to have the highest risk of myopathy compared to other races [5].

SINAM is an extremely rare and severe complication of statin use that presents as rapidly progressing symmetrical proximal muscle weakness and dysphagia with significantly elevated serum CK levels (usually >3,000 U/L) that does not resolve with discontinuation of the drug [6]. Biopsy results typically show necrotic and regenerating myofibers with minimal or no inflammatory infiltrates [7]. SINAM is known to involve the development of autoantibodies against HMGCR (first identified in 2010), which are used to distinguish it from other causes of necrotizing autoimmune myopathy [8]. HMGCR is the rate-limiting enzyme in cholesterol synthesis and is also the target site of statin inhibition; it is known to have increased levels in muscle cells exposed to statins [9]. Although the pathophysiology remains unclear, multiple immune processes are believed to contribute. There is also believed to be a genetic predisposition to the condition, suggested by the increase incidence of DRB1*11:01 in patients with positive anti-HMGCR antibody [10]. Although most cases reported have been due to atorvastatin (as opposed to other statins), an association has not been established [11]. 

A number of similar cases have been reported in recent years. Often times, the presenting symptoms mimicked other more common autoimmune myopathies such as polymyositis [11]. A systematic literature review of 100 SINAM cases by Nazir et al. found that the mean duration of statin use prior to the onset of myopathy symptoms was 40.48 months, which is much longer than our patient’s presentation after only one month of use [12]. Treatment outcomes for SINAM patients were favorable, with 91% of patients having a complete resolution of symptoms. However, methods of treatment varied greatly. Remission was induced by steroids alone in only 8.82% of the cases; most patients required azathioprine, IVIG, or plasmapheresis. The majority of patients (83.82%) required two or more immunosuppressants. In comparison to these studies, our patient had a protracted course of illness despite early detection in atorvastatin course, and showed no significant improvement despite a wide array of treatments attempted.

## Conclusions

SINAM is a recently described subset of myopathy and severe potential side effect of statin use that should be suspected in patients who develop persistent progressive muscle weakness and CK elevation despite discontinuation of the drug. Due to the similarity of its presentation to other causes of inflammatory myopathy and rhabdomyolysis, SINAM is often difficult to diagnose. It is important for clinicians to be aware of this condition and its presenting symptoms. Appropriate antibody testing including anti-HMGCR and muscle biopsy should be undertaken in suspected patients to avoid a misdiagnosis and delay in treatment onset. Further research is needed to better elucidate optimal management of this rare condition.
